# Short-Term Effects of a Ready-to-Drink Pre-Workout Beverage on Exercise Performance and Recovery

**DOI:** 10.3390/nu9080823

**Published:** 2017-08-01

**Authors:** P. Blaise Collins, Conrad P. Earnest, Ryan L. Dalton, Ryan J. Sowinski, Tyler J. Grubic, Christopher J. Favot, Adriana M. Coletta, Christopher Rasmussen, Mike Greenwood, Richard B. Kreider

**Affiliations:** 1 Exercise and Sport Nutrition Lab, Human Clinical Research Facility, Texas A&M University, College Station, TX 77843, USA; blaise_collins@tamu.edu (P.B.C.); conradearnest@exchange.tamu.edu (C.P.E.); ryanldalton@exchange.tamu.edu (R.L.D.); ryansowinski6@gmail.com (R.J.S.); tylergrubic@tamu.edu (T.J.G.); cfavot@tamu.edu (C.J.F.); AMColetta@mdanderson.org (A.M.C.); crasmussen@tamu.edu (C.R.); mgreenwood26@tamu.edu (M.G.); 2 Nutrabolt, Bryan, 3891 S. Traditions Drive, Bryan, TX 77807, USA

**Keywords:** resistance training, dietary supplement, sport nutrition, ergogenic aid

## Abstract

In a double-blind, randomized and crossover manner, 25 resistance-trained participants ingested a placebo (PLA) beverage containing 12 g of dextrose and a beverage (RTD) containing caffeine (200 mg), β-alanine (2.1 g), arginine nitrate (1.3 g), niacin (65 mg), folic acid (325 mcg), and Vitamin B12 (45 mcg) for 7-days, separated by a 7–10-day. On day 1 and 6, participants donated a fasting blood sample and completed a side-effects questionnaire (SEQ), hemodynamic challenge test, 1-RM and muscular endurance tests (3 × 10 repetitions at 70% of 1-RM with the last set to failure on the bench press (BP) and leg press (LP)) followed by ingesting the assigned beverage. After 15 min, participants repeated the hemodynamic test, 1-RM tests, and performed a repetition to fatigue (RtF) test at 70% of 1-RM, followed by completing the SEQ. On day 2 and 7, participants donated a fasting blood sample, completed the SEQ, ingested the assigned beverage, rested 30 min, and performed a 4 km cycling time-trial (TT). Data were analyzed by univariate, multivariate, and repeated measures general linear models (GLM), adjusted for gender and relative caffeine intake. Data are presented as mean change (95% CI). An overall multivariate time × treatment interaction was observed on strength performance variables (*p* = 0.01). Acute RTD ingestion better maintained LP 1-RM (PLA: −0.285 (−0.49, −0.08); RTD: 0.23 (−0.50, 0.18) kg/kg_FFM_, *p* = 0.30); increased LP RtF (PLA: −2.60 (−6.8, 1.6); RTD: 4.00 (−0.2, 8.2) repetitions, *p* = 0.031); increased BP lifting volume (PLA: 0.001 (−0.13, 0.16); RTD: 0.03 (0.02, 0.04) kg/kg_FFM_, *p* = 0.007); and, increased total lifting volume (PLA: −13.12 (−36.9, 10.5); RTD: 21.06 (−2.7, 44.8) kg/kg_FFM_, *p* = 0.046). Short-term RTD ingestion maintained baseline LP 1-RM (PLA: −0.412 (−0.08, −0.07); RTD: 0.16 (−0.50, 0.18) kg/kg_FFM_, *p* = 0.30); LP RtF (PLA: 0.12 (−3.0, 3.2); RTD: 3.6 (0.5, 6.7) repetitions, *p* = 0.116); and, LP lifting volume (PLA: 3.64 (−8.8, 16.1); RTD: 16.25 (3.8, 28.7) kg/kg_FFM_, *p* = 0.157) to a greater degree than PLA. No significant differences were observed between treatments in cycling TT performance, hemodynamic assessment, fasting blood panels, or self-reported side effects.

## 1. Introduction

Ready-to drink (RTD) pre-workout supplements and energy drinks have been purported to improve exercise performance and/or cognitive function [1,2,3]. These supplements typically contain combinations of various purported ergogenic nutrients including carbohydrate, caffeine, amino acids, creatine, beta-alanine, vasodilators (e.g., nitrates, l-citrulline, l-arginine), nutrients purported to improve concentration (e.g., citicoline), and various vitamins [3,4,5,6]. Recent position stands from the International Society of Sports Nutrition (ISSN) concluded that consuming energy drinks primarily containing caffeine and beta alanine can approve acute exercise performance, cognitive function, and/or training adaptations [3,7,8]. More recently, ingestion of nitrates prior to exercise has been reported to improve endurance exercise efficiency and high-intensity exercise performance [9,10,11,12,13]. Consequently, there has been interest in examining the individual and synergistic effects of ingesting pre-workout supplements and/or energy drinks prior to exercise and during training [3,6]. Additionally, to assess the acute and short-term safety of adding these types of supplements to the normal diet, at the absolute doses recommended, as this is the typical way consumers take these supplements. This study examined the use of an RTD version of a market leading pre-workout supplement containing caffeine anhydrous (200 mg), beta-alanine (2.1 g), arginine nitrate (1.3 g), niacin (65 mg), folic acid (325 mcg) and cobalamin (45 mcg) on indices of muscular strength and endurance.

In brief, caffeine has been shown to improve mental acuity, maximal strength, maximal power, and muscular endurance when taken acutely at doses of 3 to 9 mg/kg [7,14,15,16,17,18]. For example, doses as low as 5 mmol of nitrates (310 mg) have been shown to enhance sprint performance and increase time to fatigue [19,20,21]. β-alanine has been reported to improve strength and endurance performance typically when ingested at doses of 4 to 6 g/day for several weeks [8]. Similarly, l-arginine has been reported to activate the nitric oxide synthase pathway, resulting in vasodilation and enhanced blood flow to working tissues [22]. However, less is known about the combination of arginine and nitrate [23,24,25,26,27]. The rationale of combining these two ingredients is to enhance the bioavailability of l-arginine; hence potentially increasing an overall ergogenic effect [6]. 

Recent work from our group has demonstrated that combining various combinations of caffeine, nitrate, creatine, and β-alanine as part of a pre-workout powdered drink formula had some positive effects on cognitive and/or exercise performance [28,29,30]. The aim of this study was to examine the effects of consuming a “ready-to-drink” (RTD) version of a market leading pre-workout supplement. Our primary outcome was the assessment of exercise performance recovery after acute and short-term supplementation, while the secondary outcome was assessment of acute and short-term safety. We hypothesize that the RTD studied would improve resistance-exercise performance recovery and 4 km cycling time-trial (TT) performance without undue alterations in hepatorenal and muscle enzyme function, hemodynamic response to a hemodynamic challenge, or self-reported side effects.

## 2. Materials and Methods

### 2.1. Study Overview

Prior to starting the study, approval was obtained from the Texas A&M University Institutional Review Board (#2016-754F). Although not required, we also registered the study with clinicatrials.gov (#NCT03032549). Recreationally active men and women between the ages 18–40 years were recruited to participate in this study through the campus email system as well as posting flyers throughout the university. Participants responding to recruitment advertisements were initially screened by phone to determine general eligibility. Inclusion criteria required that each participant have at least six months of resistance training experience immediately prior to entering the study, inclusive of bench press and leg press or squat training. Participants were excluded from participation if they had a history of treatment for metabolic disease (i.e., diabetes), hypertension, hypotension, thyroid disease, arrhythmias, and/or cardiovascular disease; if they were currently using any prescription medication with the exception of birth control; if they were pregnant, lactating, or planned to become pregnant within the next month; if they had a history of smoking; if they drank excessively (>12 drinks per week); or, if they had a recent history of consuming dietary supplements or energy drinks containing β-alanine or high amounts of caffeine within eight weeks of the start of supplementation. 

**Figure 1:**
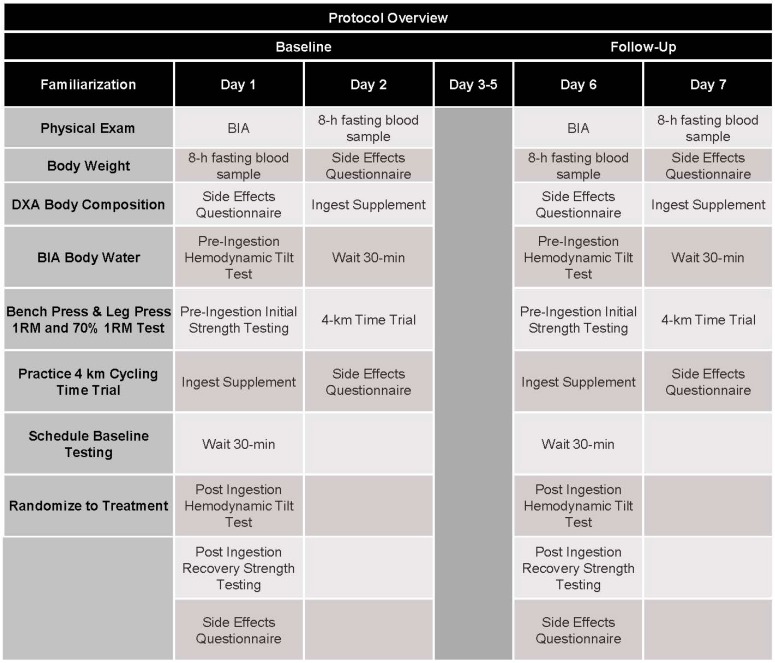
Protocol Overview.

Figure 1 presents the general study design. Participants meeting initial phone screening conditions were invited to attend a familiarization session. During the familiarization session, participants signed informed consent statements and had a physical exam inclusive of providing their medical history, determination of resting heart rate and blood pressure, and assessment of body composition via dual-energy X-ray absorptiometry (DXA) and bioelectrical impedance (BIA). Once cleared to participate, participants had bench one-repetition maximum (1-RM) determined, performed 3 sets of 10 repetitions on the bench press at 70% of 1-RM, with the last set completed to failure. Participants followed a similar familiarization on the leg press and then rested for 15 min prior to performing a warm-up and a 4 km TT on an electronically-braked cycle ergometer. Participants were then randomized to initiate the study with their respective treatments.

Baseline testing took place on two days. Day 1 included fasting blood, hemodynamic assessment, and strength testing while Day 2 included the 4 km TT. All fasting blood samples were obtained following an 8 h fast primarily between the hours of 0600–0900. Participants performed a pre-supplementation hemodynamic postural challenge test using a tilt table, performed 1-RM and a muscular endurance test (3 sets of 10 repetitions with the last set to failure) on the bench press and leg press. Participants then ingested their assigned RTD, waited 15 min and were placed in the supine position on the tilt table for 15 min prior to performing the postural hemodynamic challenge test. Participants then repeated the 1-RM test and one-set to failure at 70% of 1-RM on the bench press and leg press to assess recovery. The rationale for this approach was to determine whether ingestion of the RTD would influence exercise capacity after exhaustive exercise and toward the end of a training session. On Day 2, participants ingested the assigned treatment, waited 30 min, performed a standard warm-up, and performed a 4 km cycling TT. Participants continued the supplementation protocol for Days 3 to 5 and then returned to the laboratory for follow-up testing on Day 6 and Day 7 to repeat experiments as described. 

### 2.2. Supplementation Protocol

Participants were instructed to maintain normal training, diet, and caffeine intake habits throughout the study. Participants were assigned in a randomized, double-blind, cross-over manner to a placebo (PLA) beverage containing 6.0 g dextrose and non-caloric sweetener or a beverage (RTD) containing caffeine anhydrous (200 mg), β-alanine (2.1 g), niacin (65 mg), folic acid (325 mcg), Vitamin B12 (45 mcg), arginine nitrate (1.3 g providing about 350 mg of nitrates and 950 mg of arginine). A 7 to 10 days washout period was observed between treatment experiments consistent with prior research on caffeine and nitrates using crossover designs. The beverages were prepared by a third party (South East Bottling and Beverage, Dade City, FL, USA) in 10 oz. of purified water matched for color and flavor in indistinguishable bottles. The nutrient contents of the RTD’s were analyzed for contaminants and nutrient content by Century Foods International (Sparta, WI, USA). The pre-packaged bottles were received in boxes containing sealed bottles generically labeled as “Treatment A” and “Treatment B” for double-blind administration. The supplement code was maintained in a sealed envelope and was not disclosed to the researchers until the completion of the study for statistical analysis. 

### 2.3. Test Methodology

#### 2.3.1. Anthropometry & Body Composition

Standardized anthropological testing included assessments for body mass and height on a Healthometer Professional 500KL (Pelstar LLC, Alsip, IL, USA) self-calibrating digital scale with an accuracy of ±0.02 kg. Total body water was determined under standardized conditions using an ImpediMed DF50 bioelectrical impedance analyzer (ImpediMed, San Diego, CA, USA). Whole body bone density and body composition measures (excluding cranium) were determined with a Hologic Discovery W Dual-Energy X-ray Absorptiometer (Hologic Inc., Waltham, MA, USA) equipped with APEX Software (APEX Corporation Software, Pittsburg, PA, USA) by using standardized procedures [31,32]. Mean test-retest reliability studies performed on male athletes in our lab over repeated standardized assessment procedures have demonstrated coefficients of variation for total bone mineral content and total fat free/soft tissue mass of 0.31–0.45% with a mean intraclass correlation of 0.985 [32]. On the day of each test, the equipment was calibrated following the manufacturer’s guidelines. 

#### 2.3.2. Blood Collection Procedures

Participants provided an 8 h fasted blood sample via venipuncture of an antecubital vein in the forearm in accordance with standard phlebotomy procedures. Approximately 10 mL of whole blood was collected at the beginning of each testing day, in one 7.5 mL BD Vacutainer^®^ serum separation tube (Becton, Dickinson and Company, Franklin Lakes, NJ, USA) and in one 3.5 mL BD Vacutainer^®^ K_2_ EDTA tube (Becton, Dickinson and Company, Franklin Lakes, NJ, USA). Both tubes sat at room temperature for 15 min, then the 7.5 mL serum separation tube was centrifuged at 3500-rpm for 10 min using a 4 °C refrigerated bench top ThermoScientific Heraeus MegaFuge 40R Centrifuge (Thermo Electron North America LLC, West Palm Beach, FL, USA). Both tubes were stored at 4 °C for 3 to 4 h prior to analysis or storage. Serum was stored at −80 °C in polypropylene microcentrifuge tubes for later analysis.

#### 2.3.3. Blood Chemistry

Blood serum samples were analyzed for the following: alkaline phosphatase (ALP), aspartate transaminase (AST), alanine transaminase (ALT), creatinine, blood urea nitrogen (BUN), creatine kinase (CK), lactate dehydrogenase (LDH), glucose, total cholesterol, high density lipoprotein (HDL), low density lipoprotein (LDL), and triglycerides (TG) using a Cobas^®^ c111 (Roche Diagnostics, Basel, Switzerland) automated clinical chemistry analyzer. The Cobas^®^ c111 automated clinical chemistry analyzer was calibrated daily per manufacturer guidelines. This analyzer has been known to be valid and reliable in previously published reports [33]. The internal quality control for the Cobas^®^ c111 is performed using two levels of control fluids purchased from the manufacturer to calibrate acceptable standard deviation (SD) and coefficient of variation (C_V_) values for all assays. Samples were re-run if the values observed were outside control values and/or clinical norms according to standard procedures. Prior analysis in our lab has yielded test-to-test reliability of a range of CV from 0.4 to 2.4% for low control samples and 0.6–1.9% on high controls. Precision has been found between 0.8 and 2.4% on low controls and 0.5–1.7% on high controls.

#### 2.3.4. Hemodynamic Challenge Test

During the strength testing days (Days 1 and 6) participants had hemodynamic response assessed at two time points, prior to initial strength testing measures and following supplementation. Participants were placed on a standard tilt table in a supine position (Gravity 4000 Inversion Table; City of Industry, CA, USA). After 15 min, blood pressure and heart rate were assessed and recorded. Next, the tilt table was adjusted to vertical where the participant rested for 2 min and the metrics were re-assessed. Participants then performed pre-supplementation muscular strength and endurance tests, ingested the assigned treatment, and rested for 15 min prior to be placing on the tilt table in the supine position for 15 min. Heart rate and blood pressure measurements were then taken prior to and after 2 min of being moved to a vertical position. Mean arterial pressure was calculated as ((2 × DBP) + SBP)/3 as an indicator of venous return. Rate pressure product (RPP) was calculated as the product of heart rate times systolic blood pressure and represents an indirect assessment of myocardial oxygen demand. We chose these latter two tests as they represent a more robust response to a cardiovascular challenge compared to heart rate or blood pressure alone. Hemodynamic response was defined as the change in systolic blood pressure, diastolic blood pressure, heart rate, mean arterial pressure and rate pressure product from the supine to upright position.

#### 2.3.5. Self-Reported Side Effects

The side effect questionnaires were completed before and after each testing session to access perceived side effects and monitor compliance with the supplementation protocol. The questionnaires were completed a total of 16 times by each participant over the duration of the study: two times each testing day for four testing days per supplement for two different supplements. Participants were asked to rank the frequency and severity of their symptoms—dizziness, headache, tachycardia, heart skipping or palpitations, shortness of breath, nervousness, blurred vision, and unusual or adverse effects. Participants were asked to rank their perception of symptoms using the following scale: 0 (none), 1 (minimal: 1–2/week), 2 (slight: 3–4/week), 3 (occasional: 5–6/week), 4 (frequent: 7–8/week), or 5 (severe: 9 or more/week).

#### 2.3.6. Strength Testing

Participants performed three warm up sets prior to performing 1-RM attempts (i.e., one set of 10 at 50%, one set of 5 at 70%, and one set of 3 at 90% of anticipated 1-RM). Following the warm-up, participants gradually increased weight between 1-RM attempts until they could not lift the load under their own volition. Following determination of 1-RM, participants performed two sets of 10 repetitions with 2 min rest recovery between sets at the closest bar/leg press weight corresponding to 70% of familiarization session 1-RM. Participants then rested 2 min and performed a third set to failure. After 2 min of rest, participants followed the same procedure to determine leg press 1-RM and leg press muscular endurance. Hand placement on the bench press bar and seat and foot positioning on the leg press were placed in the same position among attempts and testing sessions. 

The initial strength tests were performed to pre-fatigue the participant before assessing recovery performance after RTD ingestion. The recovery muscular strength and endurance performance assessment involved performing a 1-RM test and then one set to failure at 70% of the familiarization 1-RM following similar procedures as described above. In this way, the effects of acute RTD ingestion could be assessed on muscular strength and endurance recovery following a standard bout of resistance exercise on Day 1, the effects of 6 days of RTD ingestion could be assessed on initial muscular strength and performance on Day 6, and the effects of acute RTD ingestion on recovery of exhaustive exercise could be assessed after 6 days of supplementation on Day 6. We did not have the participants repeat their performance of 2 sets of 10 repetitions during recovery analysis, as the initial bout of exercise fatigued the participants, it was unlikely participants could complete all 10 repetitions of these sets, and it was unnecessary to assess recovery muscular endurance. 

Total 1-RM weight lifted in kg and the number repetitions performed each set using 70% of the familiarization weight (rounded to the nearest 2.27 kg or 5 lbs. that could be put on the bar) were recorded. Total lifting volume was calculated by multiplying the 70% of 1-RM weight lifted times the number of repetitions performed each set and summing the total volume performed for all sets. Total combined lifting volume was calculated by adding the bench press and leg press total lifting volumes. Day to day test reliability of performing this performance test in our lab on resistance-trained participants has yielded a CV of 0.34 and an intraclass correlation coefficient of 0.99 for three sets of bench press total lifting volume and an intraclass correlation coefficient of 0.96 for three sets of leg press total lifting volume.

#### 2.3.7. Time-Trial Performance

Time-trial performance was examined on a magnetically braked cycle ergometer (Lode Sport Excalibur, Groningen, The Netherlands) over a distance of 4 km. Participants were allowed a one minute warm up with a gradually increasing load. At the completion of the warm up, a standardized resistance (4 J/kg/rev) was applied and the participant was instructed to complete the distance in as short a time as able. Upon completion, the participant was instructed to continue at a slow pace to facilitate recovery. Data were recorded as time to completion and average power in Watt.

### 2.4. Statistical Analysis

Data were analyzed using IBM^®^ SPSS^®^ Version 24 software (IBM Corp., Armonk, NY, USA). The sample size was determined based on the expectation of a five percent improvement in exercise performance and corresponding power of 0.80. The analysis was initiated by inspecting data for missing values using Little’s test for data missing completely at random (MCAR). This analysis showed the data were MCAR (*p* = 1.0, <1.5%) and subsequently replaced using a multiple imputation algorithm. Data were then analyzed using univariate, multivariate and repeated measures general linear models (GLM) using gender and relative caffeine intake (mg/kg) as covariates using the following models. 

Model 1. The cohort was examined for potential gender-by-treatment effects, finding none. Hence, the data were pooled into one cohort instead of reporting gender data separately. 

Model 2. Since menstrual cycle, birth control medications, and other gender-related parameters were not controlled, gender was included as a covariate. 

Model 3. Given the weight difference between males and females in the study, we further adjusted our analysis for relative caffeine intake (mg/kg). Herein, we present the results for Model 3 with performance-related data expressed in absolute and relative terms to fat free mass.

Data were also examined for a treatment order effect to confirm that randomization procedures were effective. Least significant difference post hoc comparisons were used to compare between-treatment differences when significant time × treatment interaction effects were observed. Hematological variables were also examined relative to normal clinical limits to examine the frequency of changes in hematology outside of normal, clinical limits from baseline to follow-up using a Chi-square and adjusted residual analyses. This analyses examined the likelihood of excursions outside of clinical limits for each treatment as follows: (1) No change; (2) Normal at Baseline, High at Follow-up; (3) High at Baseline, High at Follow-up; (4) High at Baseline, Normal at Follow-up. Data are reported as mean (SD), mean change from baseline and 95% confidence intervals, and frequency of occurrence according to the chi-square analysis. Data were considered statistically significant when the probability of type I error was 0.05 or less while tendencies towards statistical significance were noted when *p*-levels were *p* > 0.05 to *p* < 0.10. 

## 3. Results

### 3.1. Participants

Thirty-one individuals initially signed informed consent prior to data collection; however, five individuals dropped out prior to baseline testing due to schedule or personal reasons. Twenty-six participants began the study, with one male dropping out after the first baseline session due to time constraints. Data from a total of 25 participants were included in statistical analysis. Participant demographic data are presented in Table 1. These data demonstrate that the participants were recreationally active resistance-trained individuals and that participants differed based on gender on a number of variables. No time × gender × treatment interactions were observed on variables evaluated or relative caffeine intake effect. Further, the fully adjusted statistical model did not produce a substantial difference to the unadjusted model. Nevertheless, since menstrual cycle and birth control medication was not controlled, results were adjusted via covariate analysis for gender and relative caffeine intake.

### 3.2. Performance

Table 2 presents muscular strength and performance results normalized to fat free mass (FFM). Multivariate analysis revealed a significant overall Wilks’ Lambda treatment × time interaction effect (*p* = 0.01). Univariate analysis revealed significant treatment × time interactions in bench press (*p* = 0.04) and leg press repetitions to failure (*p* = 0.04) while bench press lifting volume (*p* = 0.09) and total combined lifting volume (*p* = 0.09) tended to interact. Post-hoc analysis revealed that acute RTD ingestion on Day 1 significantly improved recovery bench press muscular endurance to a greater degree than following PLA ingestion. Pair-wise differences were also observed between treatments in Day 1 recovery leg press endurance (*p* = 0.01) and tended to improve leg press lifting volume (*p* = 0.054). No significant differences were observed between groups in follow-up assessments. Similar findings were observed when analyzing absolute performance results. No significant differences were observed among treatments in cycling performance time or average power output expressed in absolute (*W*) or relative (W/kg_FFM_) terms (Table 3).

**Figure 2:**
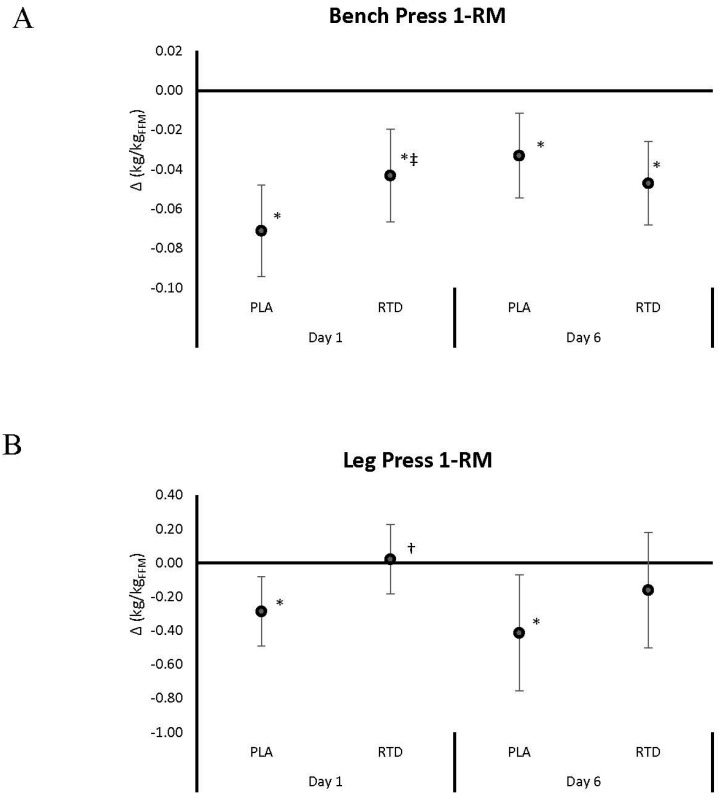
Data present mean change (95% CI) in bench press (Panel **A**) and leg press (Panel **B**) one repetition maximum (1-RM) from baseline. Confidence intervals not crossing zero are statistically significant (*p* < 0.05). * Represents *p* < 0.05 difference from baseline, † represents *p* < 0.05 difference between treatments. ‡ Represents *p* < 0.05 to 0.10 tendency towards significance between treatments.

**Figure 3:**
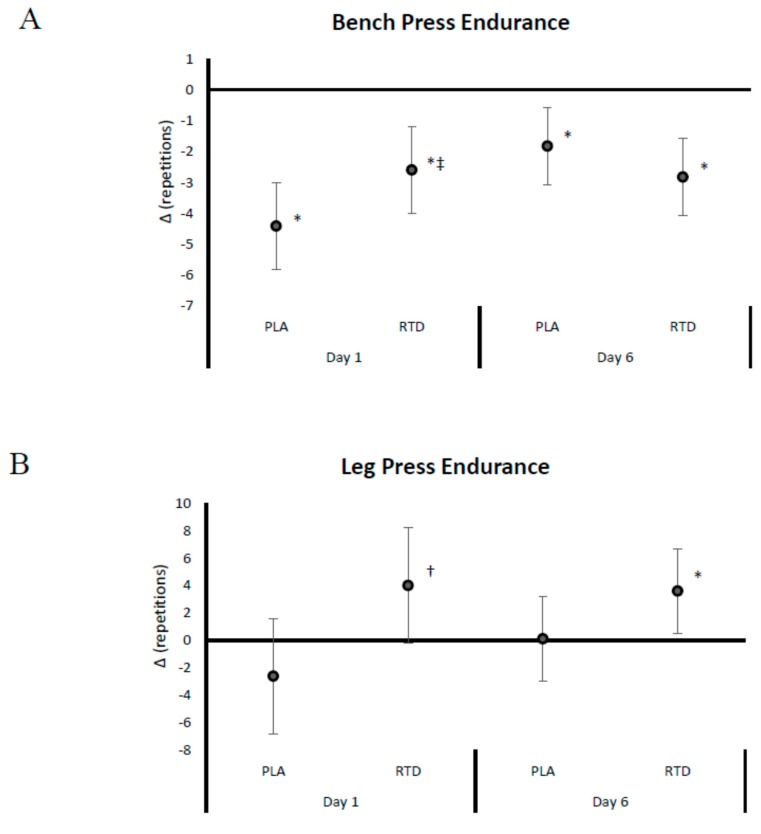
Data present mean change (95% CI) in bench press (**A**); and leg press (**B**) muscular endurance repetitions to failure at 70% of one repetition maximum (1-RM) from baseline. Confidence intervals not crossing zero are statistically significant (*p* < 0.05). * Represents *p* < 0.05 difference from baseline, † represents *p* < 0.05 difference between treatments. ‡ Represents *p* > 0.05 to 0.10 tendency towards significance between treatments.

**Figure 4:**
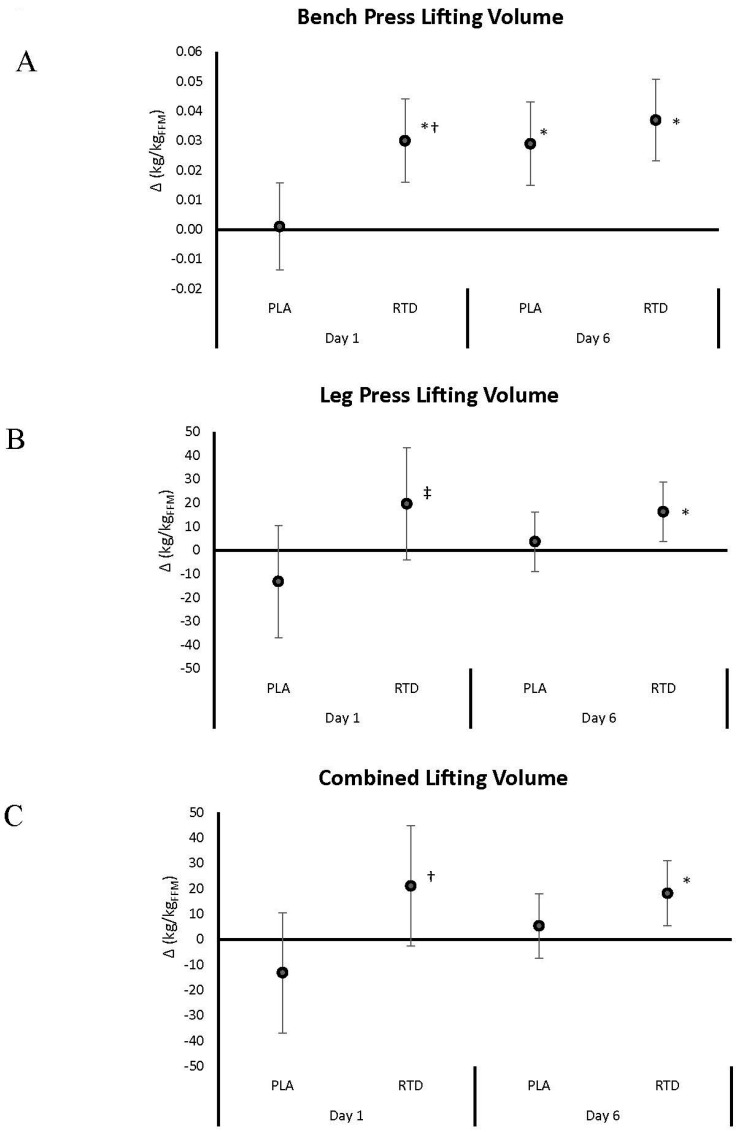
Data present mean change (95% CI) in bench press (**A**); leg press (**B**) and total (combined) lifting volume; (**C**) from baseline. Confidence intervals not crossing zero are statistically significant (*p* < 0.05). * Represents *p* < 0.05 difference from baseline, † represents *p* < 0.05 difference between treatments. ‡ Represents *p* > 0.05 to 0.10 tendency towards significance between treatments.

**Figure 5:**
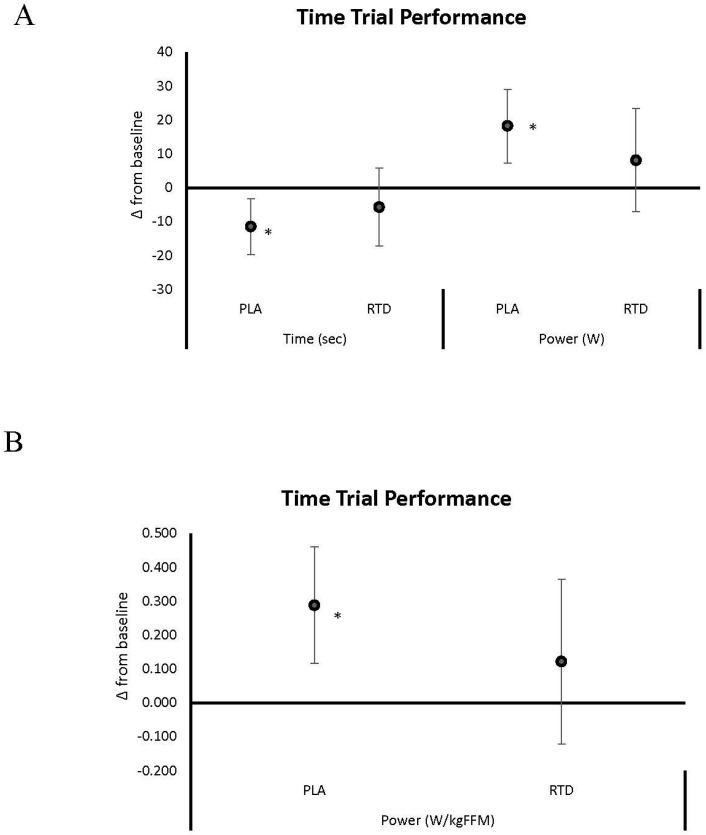
Data present mean change (95% CI) in 4 km time-trial performance from baseline expressed in completion time and absolute power output (**A**); and relative power output (**B**). Confidence intervals not crossing zero are statistically significant (*p* < 0.05). * represents *p* < 0.05 difference from baseline.

Figure 2, Figure 3, Figure 4 and Figure 5 show mean changes from baseline with 95% CI’s for 1-RM, repetitions to failure (RtF), lifting volume, and time-trial performance data, respectively. Acute RTD ingestion tended to maintain BP 1-RM to a greater degree (PLA: −0.071 (−0.09, −0.05); RTD: −0.043 (−0.05, −0.01) kg/kg_FFM_, *p* = 0.086) and maintained leg press 1-RM performance (PLA: −0.285 (−0.49, −0.08); RTD: 0.23 (−0.50, 0.18) kg/kg_FFM_, *p* = 0.30) compared to PLA (Figure 2). After 6 days of supplementation, recovery LP 1-RM significantly decreased in the PLA but not RTD treatment (PLA: −0.412 (−0.08, −0.07); RTD: 0.16 (−0.50, 0.18) kg/kg_FFM_, *p* = 0.30). As noted in Figure 3, recovery RtF on the BP tended to be greater in the RTD versus PLA treatment on Day 1 (PLA: −4.41 (−5.8, −3.0); RTD: −2.59 (−4.0, −1.19) repetitions, *p* = 0.072) while LP RtF was significantly greater than PLA (PLA: −2.60 (−6.8, 1.6); RTD: 4.00 (−0.2, 8.2) repetitions, *p* = 0.031). On Day 6, RtF on the LP was significantly increased above baseline in the RTD group but not PLA (PLA: 0.12 (−3.0, 3.2); RTD: 3.6 (0.5, 6.7) repetitions, *p* = 0.116). Bench press lifting volume (Figure 4) was significantly increased above baseline and was significantly greater than PLA (PLA: 0.001 (−0.13, 0.16); RTD: 0.03 (0.02, 0.04) kg/kg_FFM_, *p* = 0.007) while LP lifting volume tended to be greater (PLA: −13.18 (−36.9, 10.5); RTD: 19.6 (−4.1, 43.3) kg/kg_FFM_, *p* = 0.055) in the RTD treatment on Day 1. On Day 6, LP lifting volume was increased above baseline values in the RTD but not PLA treatment (PLA: 3.64 (−8.8, 16.1); RTD: 16.25 (3.8, 28.7) kg/kg_FFM_, *p* = 0.157). Recovery total lifting volume was significantly greater in the RTD treatment compared to PLA (PLA: −13.12 (−36.9, 10.5); RTD: 21.06 (−2.7, 44.8) kg/kg_FFM_, *p* = 0.046) on Day 1 and was increased above baseline while remaining unchanged with PLA treatment on Day 6 (PLA: 5.35 (−7.4, 18.1); RTD: 18.22 (5.5, 30.9) kg/kg_FFM_, *p* = 0.157). Finally, as seen in Figure 5, cycling performance times and power output improved to a greater degree in the PLA trial from baseline (PLA: −11.48 (−22.3, −1.73); RTD: −5.72 (−15.5, 4.03) s; PLA: 0.289 (0.09, 0.49); RTD: 0.122 (−0.08, 0.32) W/kg_FFM_). However, it should be noted that baseline and follow-up performance times were faster in the RTD trials than the PLA trials (see Table 3) so it cannot be concluded that the RTD promoted an ergolytic effect. 

### 3.3. Safety Analysis

Table S1 presents hemodynamic challenge results. Although some time effects were observed as expected when changing postural position, no significant overall multivariate interaction effects (*p* = 0.15) or univariate interaction effects were observed between treatments in HR, SBP, DBP, MAP, or RPP. Blood pressure and heart rate values observed remained low and were well within normal values for apparently healthy younger individuals. Similarly, no overall multivariate or univariate effects were observed among serum or whole blood markers analyzed (Tables S2–S4) or when analyzing the frequency of changes in blood parameters outside of normal clinical ranges (Table S5). Finally, as shown in Tables S6 and S7, no significant differences were observed between treatments in perceived side effects monitored (i.e., headache, dizziness, tachycardia, palpitations, dyspnea, nervousness, or blurred vision). 

## 4. Discussion

The aim of this study was to examine whether acute and/or short-term term ingestion of a commercially available pre-workout RTD beverage would affect workout performance, hemodynamic reactivity, and/or hematological affects during a 7 days intervention period. Overall, there was some evidence of better maintenance of recovery 1-RM and improvement in recovery muscular endurance with acute (Day 1) and short-term (Day 6) RTD supplementation. These findings suggest that acute and/or short-term ingestion of this RTD beverage may provide ergogenic benefit after a short recovery from resistance-training. However, ingestion of this RTD had no effects on 4 km cycling time-trial performance. Additionally, we observed no evidence that acute or short-term ingestion of this RTD negatively affected hemodynamic responses to a standardized hemodynamic challenge, fasting blood makers, or perceived side effects. Based on these findings, we accept our hypotheses that the RTD studied would improve resistance-exercise performance and recovery following pre-exhaustive exercise without undue alterations in hepatorenal and muscle enzyme function, hemodynamic responses to a postural challenge, or self-reported side effects. However, we found no evidence that acute and/or short-term ingestion of this RTD affected 4 km cycling time-trial performance in non-trained cyclists. The following discussion provides additional insight as to results observed. 

### 4.1. Performance

Caffeine is a well-known for improving cognitive and exercise performance [7,16,34]. The general recommendation is that individuals consume between 3–9 mg/kg of caffeine in order to promote ergogenic benefit in terms of exercise [7]. However, a number of studies have reported that ingestion of absolute or relative doses of caffeine in doses less than 200 mg or 3 mg/kg improved exercise performance [7,14,35,36,37,38,39,40]. Caffeine is a primary ingredient in pre-workout supplements and drinks that have also been reported to enhance cognitive and/or exercise performance [3]. For example, Souza et al. [34] performed a meta-analysis of caffeine-containing energy drinks and reported that consumption of these products promoted significant improvements in muscle strength and endurance (ES = 0.49), endurance performance (ES = 0.53), jumping (ES = 0.29) and sport-specific actions (ES = 0.51), but not in sprinting (ES = 0.14). 

In the present study, participants consumed 200 mg of caffeine providing relative caffeine intake of 2.51 ± 0.4 mg/kg for males and 3.1 ± 0.5 mg/kg for the females. While the relative doses of caffeine contained in the RTD studied were slightly less than recommendations, we found that ingesting this RTD prior to exercise (acute) and/or for 6 days (short-term) promoted better maintenance of 1-RM strength and muscle endurance. These findings support our prior reports [28,29,30] as well as previous studies reporting ergogenic benefits of consuming caffeine containing energy drinks on exercise and/or cognitive performance [14,35,36,37,38,39,40]. It is possible that since caffeine was ingested with other nutrients, there may be synergistic effects thereby reducing the need to ingest as much caffeine [3]. For example, Souza and colleagues [34] reported that consuming energy drinks with taurine may have a greater impact on efficacy than the caffeine content. While taurine was not contained in the RTD studied in the present study, results provided evidence of some ergogenic benefit. However, not all studies have reported that ingestion of caffeine containing pre-workout supplements improve performance. For example, Hendrix et al. [41] examined the effects of ingesting a pre-workout supplement containing 400 mg of caffeine, 67 mg of capsicum, and 10 mg of bioperine on performance. The researchers found that ingestion of the pre-workout supplement had no effect on bench or leg press 1-RM or time to exhaustion when cycling at 80% of maximal power output. 

A number of studies have reported that ingestion of about 300 mg of nitrates prior to exercise can improve exercise performance [6,13,17]. Most of the initial research on nitrate supplementation focused on the impact of nitrates on improving submaximal exercise efficiency [17,18,42,43]. However, there has been more recent interest in examining the effects of nitrate supplementation on high-intensity intermittent exercise performance [9,10,11,44,45,46,47,48]. These studies generally demonstrate that nitrate supplementation prior to exercise can affect endurance and high-intensity intermittent exercise performance. For this reason, addition of nitrates to pre-workout supplements have also been of interest [9,12,23,28,29,30,46,49,50,51,52,53]. Results of the present study support prior reports indicating that acute and/or short-term ingestion of supplements containing nitrates prior to exercise can affect muscular strength and/or endurance. However, in contrast to recent findings [9,12,23,29,46,51,52,53], we did not find that ingesting the RTD containing nitrates prior to exercise enhanced short duration time-trial performance. The contrasting results may be related to greater variability in studying non-trained cyclists, differences in the dosages and/or timing of ingestion of the nitrate containing RTD, or use of arginine nitrate rather than other forms of nitrates. 

### 4.2. Safety

The primary concerns related to ingesting pre-workout supplements containing caffeine and/or nitrates is the potential safety impact on cardiovascular and hemodynamic responses to exercise [3,54,55,56,57]. This was the primary reason for testing hemodynamic reactivity prior to and following resistance training exercise, as participants regularly move from a supine to standing position and, as such, could be prone to reactive hypotension accompanying RTD supplementation during exercise. In the present study, we found no evidence that ingestion of the RTD study adversely affects heart rate or blood pressure responses to a standardized postural challenge. Accordingly, we found no evidence to suggest that acute and/or short-term ingestion of this RTD significantly affected the hematological variables studied or the incidence of self-reported side effects. Consequently, the acute and short-term use of this RTD appears to be safe within the dosages and manner it was assessed in the current study. These findings are consistent with our prior studies with supplementation periods as long as 8 weeks [28,29,30,52], as well as other similar studies [58,59]. 

### 4.3. Strengths and Limitations 

A strength of our study is that we used a fairly large cohort comprised of men and women who ingested their respective treatments in addition to their normal diet in a randomized double blind, cross-over manner. Additionally, our protocol was vigorous with regard to the number of exercises performed during testing and applicable as typical resistance-training sessions are comprised of multiple sets of multiple exercises. Thus, the design used allowed for a practical assessment of the ability of resistance-trained participants to maintain performance throughout a rigorous workout. A strength of this study was also that we examined a mixed cohort of men and women in a crossover manner. While this does not discount the possibility that gender differences may exist when using a larger or single-gender study protocol, we used gender as a covariate to account for gender differences. Finally, a strength of this study was that we made a concerted effort to examine several parameters associated with safety by examining potential hemodynamic changes accompanying supplementation and exercise as well as a thorough analysis of hepatorenal and muscle enzyme function associated with the supplementation protocol. Assessment of the cardiovascular and hemodynamic responses to a postural challenge represents a similar pattern of movement as would take place during resistance training, as athletes often go from supine to standing positions throughout a workout and may experience orthostatic hypotension. Moreover, another strength of this study was that we extended the traditional reporting schema of most trials to include potential changes out of normal clinical ranges, without adverse consequence. 

Potential limitations in our study included the utilization of recommended absolute serving sizes rather than relative doses to body weight or fat-free mass. It is possible that more consistent performance results would have been observed if relative doses were used. However, this is not how these types of supplements are consumed so we decided to use normal serving sizes and control for this limitation by using relative caffeine intake as a covariate in our analysis. Additionally, although we have examined the effects of ingesting pre-workout supplements for up to 8 weeks, this study only assessed the acute and short-term effects. It is possible that the ergogenic benefits may lessen with longer periods of supplementation due to habituation, but research in this arena is limited. However, RTD products are marketed as having an immediate effect on performance without requiring a loading period or alterations in diet, so we feel this design was a practical analysis of how individuals may use this type of supplement. Another potential limitation was that we examined the effects of this RTD on recreationally-active resistance-trained participants. While this population was well-prepared to assess changes in muscular strength and endurance performance, they were not trained cyclists accustomed to performing sprints. Thus, it is conceivable that the lack of effect observed on 4 km cycling time-trial performance may have been affected by a lack of familiarity with cycling, regardless of partaking in a familiarization session. Additional research should examine whether ingestion of this type of RTD may affect sprint and/or high-intensity short-duration sprint performance. 

## 5. Conclusions

Within the limitations of the study, results indicate that the RTD studied provided some ergogenic benefit on recovery from resistance exercise with no apparent side effects observed. However, consumption of this RTD beverage did not affect 4 km cycling TT performance among non-cycling trained participants. Additional research should assess the safety and efficacy of nutrients found in pre-workout supplements so that active individuals can make an informed decision about the whether they should or should not use them during training and/or competition. 

## Figures and Tables

**Table 1:** Baseline Demographics.

Measurement	Male (*n* = 12)	Female (*n* = 13)	Overall (*n* = 25)	
	Mean SD	Mean SD	Mean SD	*p*-Values
Age (year)	23.3 ± 4	24.5 ± 4	23.9 ± 4	0.43
Height (cm)	177 ± 7	166 ± 5	171 ± 8	0.00
Weight (kg)	81.7 ± 13	65.1 ± 8	73.1 ± 13	0.00
Body Mass Index (kg/m^2^)	26.0 ± 4	23.7 ± 3	24.8 ± 4	0.12
Body Fat (%)	17.2 ± 6	28.4 ± 6	23.0 ± 8	0.00
Fat Free Mass (kg)	67.1 ± 9	47.8 ± 8	57.1 ± 13	0.00
Bench Press 1RM (kg)	88.3 ± 27	37.9 ± 10	62.1 ± 32	0.00
Bench Press 1RM (kg/kg_FFM_)	1.31 ± 0.4	0.80 ± 0.2	1.05 ± 0.4	0.00
Leg Press 1RM (kg)	455 ± 175	284 ± 89	366 ± 160	0.01
Leg Press 1RM (kg/kg_FFM_)	6.7 ± 2.0	6.0 ± 1.6	6.3 ± 1.8	0.18

Mean data presented as means ± SD. One-way ANOVA *p*-values listed for each variable. PLA: placebo, RTD: ready-to-drink pre-workout supplement, 1RM: one repetition maximum, FFM: fat free mass, kg/kg_FFM_: weight relative to participant fat free mass.

**Table 2:** Strength and Muscular Endurance Relative to Fat Free Mass.

Variable	Treatment	Day 1 Pre-Ingestion	Day 1 Post Ingestion	Day 6 Pre-Ingestion	Day 6 Post Ingestion	Treatment		
		Mean SD	Mean SD	Mean SD	Me SD	Mean SE	*p*-Value	
BP 1-RM (kg/kg_FFM_)	PLA	1.02 ± 0.38	0.94 ± 0.36	1.01 ± 0.36	0.97 ± 0.37	0.99 ± 0.06	Time	0.001
	RTD	1.03 ± 0.37	0.99 ± 0.35	1.04 ± 0.35	1.00 ± 0.36	1.02 ± 0.06	Trt	0.72
	Time	1.02 ± 0.37	0.97 ± 0.35 *	1.03 ± 0.35	0.99 ± 0.36 ‡		*I*	0.23
BP Repetitions to Failure @ 70% 1RM	PLA	9.96 ± 3.23	9.60 ± 3.65	10.13 ± 3.37	12.27 ± 3.22 *	10.5 ± 0.71	Time	0.38
	RTD	10.28 ± 4.37	12.32 ± 5.28 *†	10.30 ± 4.04	13.31 ± 4.86 *	11.6 ± 0.71	Trt	0.27
	Time	10.1 ± 3.81	11.0 ± 4.70	10.2 ± 3.69	12.8 ± 4.12		*I*	0.04
BP Lifting Volume (kg/kgFFM)	PLA	7.40 ± 3.77	7.46 ± 4.57	7.46 ± 3.73	9.17 ± 4.44	7.87 ± 0.67	Time	0.36
	RTD	7.45 ± 3.75	8.89 ± 4.07	7.48 ± 3.16	9.45 ± 3.54	8.32 ± 0.67	Trt	0.64
	Time	7.43 ± 3.73	8.17 ± 4.34	7.47 ± 3.42	9.31 ± 3.98		*I*	0.09
LP 1-RM (kg/kg_FFM_)	PLA	6.70 ± 1.66	6.41 ± 1.58	6.80 ± 1.38	6.38 ± 1.35	6.57 ± 0.26	Time	0.66
	RTD	6.72 ± 1.42	6.74 ± 1.41	6.83 ± 1.29	6.67 ± 1.52	6.74 ± 0.26	Trt	0.66
	Time	6.71 ± 1.53	6.57 ± 1.49	6.81 ± 1.33	6.53 ± 1.43		*I*	0.04
LP Repetitions to Failure @ 70% 1RM	PLA	21.2 ± 10.8	18.6 ± 8.4	20.8 ± 10.7	20.9 ± 11.0	20.3 ± 1.81	Time	0.78
	RTD	22.4 ± 15.1	26.4 ± 13.0 †	19.8 ± 9.31	25.1 ± 14.1	23.9 ± 1.80	Trt	0.17
	Time	21.8 ± 13.0	22.5 ± 11.6	20.3 ± 9.94	23.0 ± 12.7		*I*	0.11
LP Lifting Volume (kg/kg_FFM_)	PLA	94.3 ± 77.9	81.1 ± 44.4	88.3 ± 49.7	91.9 ± 57.9	88.9 ± 10.60	Time	0.75
	RTD	96.6 ± 66.7	116.3 ± 74.9 ‡	90.7 ± 47.6	106.9 ± 55.4	102.6 ± 10.60	Trt	0.37
	Time	95.5 ± 71.8	98.7 ± 63.5	89.5 ± 48.2	99.4 ± 56.6		*I*	0.11
Combined Lifting Volume (kg/kg_FFM_)	PLA	101.7 ± 79.0	88.6 ± 46.8	95.8 ± 50.5	101.1 ± 60.2	96.8 ± 10.87	Time	0.76
	RTD	104.1 ± 66.4	125.2 ± 75.5 †	98.2 ± 48.8	116.4 ± 57.3	111.0 ± 10.87	Trt	0.36
	Time	102.9 ± 72.2	106.9 ± 64.9	97.0 ± 49.2	108.8 ± 58.7		*I*	0.09

Values are means ± standard deviations. Multivariate analysis revealed overall Wilks’ Lambda treatment (*p* = 0.792), time (*p* = 0.010), and treatment × time (*p* = 0.010). Greenhouse-Geisser *p*-levels are reported with univariate analyses for time, treatment, and time × treatment interactions for each variable. * indicates a significant difference from initial measure, † indicates a significant between-treatment difference, and ‡ indicates a statistical trend between-treatments. BP = bench press, LP = leg pres, 1-RM = 1 repetition maximum, *R* FFM = Fat Free Mass, TBPV = Total Bench Press Volume, TLPV = Total Leg Press Volume, TCLV = Total Combined Lifting Volume, PLA = Placebo, RTD = Ready-to-drink Pre-workout supplement, Trt = Treatment, *I* = Time × Treatment interaction.

**Table 3:** Time Trial Performance.

Variable	Treatment	Day 2	Day 7	Treatment		
		Mean SD	Mean SD	Mean SE	*p*-Values	
Time (s)	PLA	296 ± 105	284 ± 104	240 ± 11	Time	0.70
	RTD	282 ± 94	276 ± 95	281 ± 11	Trt	0.56
	Time	289 ± 99	280 ± 99		*I*	0.41
Power (W)	PLA	224 ± 82	242 ± 93	235 ± 10	Time	0.12
	RTD	238 ± 85	246 ± 95	240 ± 10	Trt	0.62
	Time	231 ± 83	244 ± 82		*I*	0.26
Power (W/kg_FFM_)	PLA	3.87 ± 0.89	4.16 ± 0.94	4.01 ± 0.17	Time	0.26
	RTD	4.17 ± 0.87	4.29 ± 0.98	4.23 ± 0.17	Trt	0.38
	Time	4.02 ± 0.89	4.22 ± 0.95		*I*	0.25

Values are means ± standard deviations. Multivariate analysis revealed overall Wilks’ Lambda treatment (*p* = 0.62), time (*p* = 0.036), and treatment × time (*p* = 0.53). Greenhouse-Geisser *p*-levels are reported with univariate analyses for time, treatment, and time × treatment interactions for each variable. PLA = Placebo, RTD = Ready-to-drink Pre-workout supplement, FFM = Fat Free Mass, Trt = Treatment, *I* = Time × Treatment interaction.

## Supplementary Materials

The following are available online at www.mdpi.com/2072-6643/9/8/823/s1, Table S1: Hemodynamic Response to Postural Challenge; Table S2: Liver, Kidney, and Muscle Hematological Data; Table S3: Blood Lipids, Macronutrients, and Nitrate data; Table S4: Whole Blood Chemistry; Table S5: Change in blood markers relative to normal clinical limits from Day 1 to Day 7; Table S6: Frequency of Self-Reported Side Effects; Table S7: Severity of Self-Reported Side Effects.
